# Aortic Involvement in Antineutrophil Cytoplasmic Antibodies Vasculitis, a Coincidence or a Real Association?

**DOI:** 10.7759/cureus.9690

**Published:** 2020-08-12

**Authors:** Ezza Tariq, Katukuri Nishanth, Assam Arshid, Mohammed Miqdad, Ivan Cancarevic

**Affiliations:** 1 Medicine, California Institute of Behavioral Neurosciences & Psychology, Fairfield, USA; 2 Medicine, Nishtar Medical College, Multan, PAK; 3 Internal Medicine, California Institute of Behavioral Neurosciences & Psychology, Fairfield, USA; 4 Surgery, California Institute of Behavioral Neurosciences & Psychology, Fairfield, USA; 5 Internal Medicine, California Institute of Behavioral Neurosciences & Psychology, Fairfield, SAU

**Keywords:** aortitis, anca vasculitis, vasculitides, granulomatosis with polyangiitis, large vessel vasculitis

## Abstract

Antineutrophil cytoplasmic antibodies (ANCA)-associated vasculitis (AAV) is a type of small-vessel vasculitis. It is unusual for ANCA to involve aorta. However, multiple cases have been found where ANCA involved large vessels, particularly the aorta. Among vasculitides, aortic vasculitis is a part of Takayasu arteritis (TAK). In this review article, we tried to find the mechanism behind the aortic involvement in AAV. PubMed was used as a primary search engine, and all the available cases of aortic, as well as large-vessel involvement in ANCA-associated vasculitis, were thoroughly reviewed. Very limited data was available that could provide the mechanism behind this involvement. It is observed that ANCA-associated aortitis is more common in immunocompromised people; however, cases in previously healthy individuals have also been found. Pathogenesis of ANCA-related aortitis is different from Takayasu arteritis and is more close to ANCA-associated small vasculitis. ANCA-related aortitis involves the aorta through the same mechanism as it uses to involve small vessels. This rare manifestation of ANCA-associated vasculitis could be life-threatening but has a good prognosis if timely diagnosed and treated. ANCA-associated vasculitis must be considered as a differential diagnosis while treating a case of aortitis. We believe that there is a need to revise the classification of different types of vasculitides, and physicians should be aware of the possible overlap between different forms of vasculitides.

## Introduction and background

Vasculitides is a group of disorders characterized by inflammation of vessels of various sizes [1]. This causes diminished blood flow, resulting in tissue damage and ultimately, necrosis [1]. Vasculitides nomenclature was defined in the International Chapel Hill Consensus Conference according to their vessel sizes and was divided into three main classes: large, medium, and small [2-3]. Large-vessel vasculitis is divided into Takayasu arteritis (TAK) and giant cell arteritis (GCA) [4]. Medium-vessel vasculitis involves polyarteritis nodosa and Kawasaki disease [2-3]. Small-vessel vasculitis is further divided into antineutrophil cytoplasmic antibodies (ANCA)-associated vasculitis (AAV), characterized by the lack or small number of immune deposits in the vessel wall, and immune complex small-vessel vasculitis [2]. ANCA-associated vasculitis includes microscopic polyangiitis (MPA), granulomatosis with polyangiitis (GPA), eosinophilic granulomatosis with polyangiitis, and AAV involving single organs (AAV limited to kidney) [2,5]. Among vasculitides, aortic involvement is predominantly present in Takayasu arteritis [2,5].

GPA is a rare disease that was first described in 1930 [6]. It is a necrotizing vasculitis associated with cytoplasmic ANCA [7]. A limited form of granulomatosis with polyangiitis involves the upper respiratory tract, while a generalized form involves kidneys and/or lower respiratory tract [7]. MPA is associated with anti-myeloperoxidase antibodies and involves only the lower respiratory tract and kidney [8]. However, general features of inflammation such as fever, weight loss, peripheral neuropathy, or muscular and articular symptoms may also be present [8]. Granulomatosis with polyangiitis is characterized by granulomatous inflammation, while non-granulomatous inflammation, involving the upper and lower respiratory tract, is characteristic of MPA [5].

It is unusual for ANCA-associated vasculitis to cause aortic inflammation [1]. The way ANCA affects vessel walls is not clear; various factors such as infection, environmental factors, and genetic factors as well as certain drugs including hydralazine, propylthiouracil, minocycline, cocaine use, and alpha-1 antitrypsin deficiency (AAT) are assumed to be the possible risk factors [9-11]. It is suggested that ANCA leads to aortitis by causing vasorum vasculitis in the adventitia, which later spreads to media and intima [12]. It is also proposed that retroperitoneal arteries involvement (periaortitis) ultimately leads to aortic inflammation [13-14]. However, there is also a possibility of an overlap between Takayasu arteritis and ANCA-associated vasculitis [4,15].

In this review article, we will discuss the pathophysiology of ANCA-associated vasculitis, especially granulomatosis with polyangiitis, and aortic involvement with it. We will also talk about the management of ANCA-related aortic involvement.

## Review

Method and results

It is a narrative review article. PubMed was used as the primary search engine. To find articles with direct relevance, "ANCA" and "aortitis" MeSH keywords were used, which showed 18 articles in total. These were reduced to 12 after applying a 10-year filter. No filters related to age or sex were applied. No statistical analysis was done, and no quality assessment tools were applied. To discuss the topic further, we did extensive research on PubMed using terms "Takayasu arteritis in ANCA vasculitis," "Vasculitides," and "Aorta in granulomatosis with polyangiitis," which showed 132, 108842, and 142 articles, respectively. Articles containing at least one keyword and those published in peer-reviewed scientific journals were included, while rest were excluded. All the data were collected in an ethical manner, and all the efforts were taken to minimize plagiarism.

Presentation of aortic diseases in AAV

Aortic diseases proximal to ligamentum arteriosum are mostly non-atherosclerotic in nature, while those distal to it are mainly atherosclerotic [16]. ANCA-related aortic disease may present as an aneurysmal disease, aortic dissection, aortic rupture, aortic regurge, and even death [12]. Among AAV, aortic involvement is reported in both GPA and MPA but not in Churg-Strauss syndrome [12]. Patient presentation with constitutional symptoms may lead to misdiagnosis and, ultimately, serious outcomes in ANCA-related aortitis [12,16]. Chirinos et al. described a case of a 53-year-old woman who presented with fever, weight loss, malaise, night sweats, and recurrent pulmonary infiltrates and was diagnosed with community-acquired pneumonia [12]. It was later found that she had MPA, which led to aortic dissection and, ultimately, death [12]. Aortic involvement can manifest even before the typical features of ANCA-associated vasculitis or even in the absence of small-vessel vasculitis [13,17-20]. Sakemi et al. discussed a case of periaortic soft tissue mass extending from the thoracic aorta to bilateral common iliac arteries, in which no other findings related to ANCA-associated vasculitis were present, but the patient tested positive for p-ANCA [20]. Sieber et al. described a case of asymptomatic GPA, in which GPA was diagnosed nine months after granulomatous aortitis and aneurysm formation [17]. Carels et al. described a case of a 63-year-old man who developed GPA seven years later after having an aortic aneurysm, and at the time of GPA diagnosis, revision of the biopsy taken seven years ago showed typical necrotic granuloma of GPA in the aortic wall [13]. In another case described by Pan et al., a 28-year-old man, who initially presented with chest pain due to aortic dissection, was later diagnosed with GPA [18]. 

A possibility of aortic involvement should always be kept in mind while treating a patient with AAV as timely diagnosis and management could reduce the morbidity as well as mortality related to aortic involvement in ANCA-associated vasculitis [12].

Risk factors leading to aortitis in AAV

Risk factors leading to the pathogenesis of ANCA-related aortitis are not clear [9-11]. However, certain immunosuppressant drugs have been found as a possible risk factor [21-22]. Murakami et al. described a case of a 57-year-old man who was taking bevacizumab for lung adenocarcinoma and later developed p-ANCA-positive periaortitis [21]. Ginsberg et al. mentioned etanercept, an anti-tumor necrosis factor, as a possible cause of ANCA-associated large-vessel vasculitis [22]. Voorzaat et al. described a fatal case of aortic dissection due to ANCA, in which the patient was tested positive for AAT [23].

Very limited data is available that can provide a strong association between possible risk factors and aortitis related to ANCA-associated vasculitis. After reviewing the available studies about ANCA-related aortitis, we suggest that there is a need for extensive research to explore the possible risk factors that are responsible not only for ANCA-associated vasculitis but also for aortic involvement in it. 

Pathophysiology

Antineutrophil antibodies are found in the cytoplasmic granules of monocytes and neutrophils [24]. In small-vessel vasculitis, antibodies are formed against two types of antigens [24]. ANCA directed against anti-proteinase 3 is categorized into GPA, and those against anti-myeloperoxidase are associated with MPA [24-25]. It is proposed that antineutrophil cytoplasmic antibodies are produced against the newly exposed epitopes referred to as cryptic sites of the autoantigen, which later involves the rest of the molecule [26]. It then spreads by "epitope spreading" to involve other components of a macromolecular protein complex [26]. Another hypothesis suggests that it is the autoantigen's complementary protein that initiates the immunological process in AAV [27-28]. Since vasculitis is an antigen-related process, its pathogenesis should be T-cell dependent [28]. T cells then overproduce TNF-alpha and interferon-gamma, which are major cytokines in the GPA-related inflammatory process [28]. However, neutrophil activation, monocytes, and B-cells also play an important role [29-31]. Schildhaus et al. suggested that the mechanism through which ANCA involves large vessels is the same as for ANCA-associated small-vessel vasculitis [16]. He also proposed that ANCA involves large vessels by inflammation of intima without affecting media and adventitia [16]. According to Blockmans et al., ANCA first involves the retroperitoneal muscular arteries, which later involves aorta [14]. Vaglio et al. found fibrinoid necrosis in vasa vasorum of aorta along with small and medium retroperitoneal vessel involvement and, among 12 of his patients, three were ANCA positive [32].

From the studies analyzed above, we conclude that it is still not clear how aorta is involved in ANCA-associated vasculitis, and further clinical research should be done to find the pathophysiology of this potentially fatal manifestation of ANCA-associated vasculitis.

Possibility of overlap between large-vessel vasculitis and ANCA vasculitis

Large-vessel vasculitis is divided into GCA and TAK [4]. Antineutrophil cytoplasmic antibodies are not increased in large-vessel vasculitis [33-34]. However, there is a possibility of an overlap between large-vessel and small-vessel vasculitis [17,35-36]. Sieber et al. reported a case with an overlap of GCA and GPA in which adventitia and media of aortic walls are involved in the granulomatous process with focal destruction of medial elastic lamellae [17]. The pattern of initial involvement can help to identify ANCA-associated large vasculitis as a different entity than an overlap between large-vessel vasculitis and AAV [12]. Adventitia and media are involved first in large-vessel vasculitis without an ANCA association [12], while intima is involved before media and adventitia in ANCA-associated large-vessel vasculitis [16]. Moreover, ANCA-associated vasculitis predominantly causes perivasculitis, which is not typical of a large-vessel vasculitis [37]. Blockmans et al. described a patient with abdominal periaortitis where inflammatory tissue surrounding aorta had granulomatous vasculitis, a typical feature of granulomatosis with polyangiitis [14]. Carels et al. described a case of a 63-year-old man with aortic aneurysm, who was found to have multiple areas of necrosis in the tissue surrounding the aortic wall, which is a characteristic finding in GPA [13]. Ohta et al. also found necrotizing granulomatous inflammation in aortic walls of a 38-year-old man with granulomatosis with polyangiitis [38]. Pan et al., in a case of a 28-year-old man with GPA, found visible necrosis along with granulation tissue hyperplasia in the aortic wall [18].

Keeping in view the above-described studies, there is very little evidence of an overlap between small- and large-vessel vasculitis. We believe that aortitis in ANCA-positive patients is caused by antineutrophil cytoplasmic antibodies, rather an overlap between the Takayasu arteritis and AAV. 

Diagnosis of AAV-related aortitis

While managing a case of aortitis due to ANCA-associated vasculitis, it is important to keep other differential diagnoses of aortitis in mind, including both infectious and non-infectious causes [4]. Non-infectious causes include other auto-immune diseases, including sarcoidosis, ankylosing spondylitis, Behcet's disease, Sjogren's syndrome, while infectious diseases involving aorta include syphilis and septicemia or endocarditis leading to mycotic aneurysm [4]. The possibility of atherosclerosis should also be ruled out while treating a patient who has developed aortitis [4].

Diagnostic criteria to distinguish granulomatosis with polyangiitis from other forms of vasculitis include nasal or oral inflammation; abnormal chest X-ray showing infiltrates, nodules, or cavities; urinary sediments that include either RBC casts or more than five RBCs per high-power field; and granulomatous inflammation on biopsy [6]. Two of these four criteria should be met for diagnosing GPA [6]. Antineutrophil cytoplasmic antibodies are detected by enzyme-linked immunosorbent assay (ELISA) and indirect immunofluorescence [25]. Immunofluorescence assays are more sensitive, while ELISA is more specific and detects antibodies against specific antigens [39]. Histopathological staining is sufficient for diagnosis of AAV, but special stains and cultures should be obtained to rule out possible infectious causes [6,38]. To detect the involvement of large vessels, non-invasive techniques are used, which include a high-resolution contrast-enhanced MRI combined with magnetic resonance angiography (MRA), contrast-enhanced CT along with computed tomography angiography (CTA), color Doppler sonography, positron emission tomography (PET) with F-fluorodeoxyglucose (FDG), and digital subtraction angiography (DSA) [1]. Carels et al. used an indium-labeled leukocyte scan to detect the periaortitis and confirmed it with a CT scan [13]. Ohta et al. initially did a chest X-ray when the GPA-positive patient complained of chest pain, which showed a lateral shift of the left lung, suggesting an aortic aneurysm [38]. It was then confirmed with a chest CT scan [38].

It is very important to rule out the large-vessel vasculitis as soon as a diagnosis of AAV is made, and the physician should have a low threshold for chest CTs when a patient with ANCA-associated vasculitis complains of chest pain, as a failure to do so could lead to serious, life-threatening complications [38]. It is also important to distinguish GPA, as a cause of aortitis, from other forms of vasculitis as cyclophosphamide is needed to treat GPA [6].

Treatment of AAV-related aortic involvement

Treatment of AAV includes induction with corticosteroids or cyclophosphamide, after which maintenance is achieved with rituximab or azathioprine [1,40]. Corticosteroids only reduce morbidity and mortality in large-vessel vasculitis, but they do not cure the disease nor prevent the relapses [1]. Takenaka et al. used tocilizumab to treat ANCA-associated aortitis when the patient did not respond to corticosteroids or cyclophosphamide [41]. Pineda et al. also used a combination of methotrexate, corticosteroids, and rituximab infusion to successfully treat GPA-associated aortic inflammation [42]. Surgery is needed when a patient develops serious complications, such as an aortic aneurysm or dissection [38]. 

As summarized in Table 1, ANCA-related aortitis is a very treatable disease, and if timely diagnosed and managed, the patient can recover uneventfully [38]. Prognosis of ANCA-associated aortitis is the same as for ANCA-associated small-vessel vasculitis [16].

**Table 1:** Presentation of aortic diseases in ANCA-associated vasculitis Acronyms: male (M); female (F); hypertension (HTN); methylprednisolone (MDP); family history (F/H); chronic obstructive pulmonary disease (COPD); alpha-1 antitrypsin (AAT).

ID	Age (years) /sex	Presentation	Possible risk factors/associated features	Aortic involvement	Treatment	Prognosis/outcome
Case 1 [14]	42/M	Fatigue, night sweats, chills, weight loss, abdominal pain	Ankylosing spondylitis	Periaortitis and aortic dissection	Emergency surgery, steroids, cyclophosphamide	Slow recovery
Case 2 [16]	63/M	Weight loss, peripheral edema, cutaneous ulcers, fever	None found	Inflammation of aortic intima	Conservative treatment, antibiotics	Death
Case 3 [13]	63/M	Fatigue, fever, back pain radiating to legs. GPA was diagnosed seven years later.	Idiopathic	Aortic aneurysm	MDP, surgery	Good/p-ANCA titers disappeared
Case 4 [43]	51/M	Lower back pain, weight loss	HTN, smoking, chemotherapy	Aortic aneurysm	MDP, cyclophosphamide	Anti-PR3 titers dropped
Case 5 [38]	38/M	Chest pain, 22 years after GPA diagnosis	None found	Aortic aneurysm, rupture	Emergency surgery, prednisolone	Uneventful recovery
Case 6 [41]	47/F	Lower back and hip pain after GPA diagnosis	None mentioned	Aortitis	Prednisolone, cyclophosphamide, tocilizumab	Uneventful recovery
Case 7 [23]	70/M	Sudden severe back pain 18 months after MPA diagnosis	Non-smoker, AAT deficiency, GCA	Aortic dissection	MDP, surgery	Death
Case 8 [18]	28/M	Fatigue, chest pain	None found	Aortic dissection	Surgery, MDP, cyclophosphamide	Uneventful recovery

## Conclusions

The main purpose of this paper is to emphasize the possibility of aortic involvement in AAV. Aortic involvement in ANCA-associated vasculitis can range from aortitis to aortic aneurysm and rupture. ANCA-associated aortitis is reported in both granulomatosis with polyangiitis and microscopic polyangiitis but not in Churg-Strauss syndrome. Immunosuppression appears to be the possible risk factor. However, there is a lack of data providing any strong association between the possible risk factors and ANCA-associated aortitis. Diagnosis is made clinically and is confirmed with a biopsy. There is a need for broader research to find the mechanism behind ANCA-associated aortitis, as this will help future clinicians in the timely diagnosis and treatment of this serious manifestation of ANCA-associated vasculitis.
